# Metabolic and inflammatory profiles, gut microbiota and lifestyle factors in overweight and normal weight young thai adults

**DOI:** 10.1371/journal.pone.0288286

**Published:** 2023-07-14

**Authors:** Surasawadee Somnuk, Surat Komindr, Sudjai Monkhai, Thitirat Poolsawat, Massalin Nakphaichit, Bandhita Wanikorn

**Affiliations:** 1 Department of Sports and Health Sciences, Faculty of Sports Science, Kasetsart University, Kamphaeng Saen Campus, Nakhon Pathom, Thailand; 2 Division of Nutrition and Biochemical Medicine, Department of Medicine, Faculty of Medicine, Ramathibodi Hospital, Mahidol University, Bangkok, Thailand; 3 Wangnumkeaw Sub-district Health Promotion Hospital, Nakhon Pathom, Thailand; 4 Department of Biotechnology, Faculty of Agro-Industry, Kasetsart University, Bangkok, Thailand; Wageningen Universiteit, NETHERLANDS

## Abstract

Obesity among young adults, especially those living in developing countries is increasing. A high body mass index (BMI) is one of the major causes of several diseases worldwide, constituting an important risk factor for non-communicable diseases (NCDs). Investigations describing the relationship between BMI, clinical and gut microbiota characteristics and lifestyle factors of overweight young adults, especially from Southeast Asian countries are limited. Metabolic and inflammatory biomarkers, fecal microbiota profiles and lifestyle factors were compared between overweight Thai young adults (n = 30, mean age 33 ± 9.48) and those with normal weight (n = 30, mean age 27 ±7.50). This study was registered with the Thai Clinical Trials Registry (TCTR20220204007). Health status including body composition, fasting glucose and insulin, lipid profiles, liver and kidney function, inflammatory biomarkers, blood pressure and fecal microbiota using 16S rRNA gene sequencing data was determined. Dietary intake was assessed using a 3-day dietary record and a food frequency questionnaire (FFQ), with physical activity levels compared using the international physical activity questionnaire (IPAQ). The overweight group had significantly higher BMI, waist-hip ratio, body fat mass, % body fat, skeletal mass, triglyceride level, C-reactive protein, insulin and blood pressure, with lower levels of high-density lipoprotein cholesterol (HDL-C) and blood urea nitrogen compared to the normal weight group. Significant differences in fecal microbiota composition at the family and genus levels were observed between the two groups. In our clinical setting, we also observed that unhealthy diets with high consumption of food rich in fat and sugar, processed meat and alcohol, and physical inactivity were associated with an increased prevalence of overweight in Thai young adults. Results provided the big picture of health and lifestyle characteristics of overweight young Thai people. Young adults should be encouraged to engage in health-promoting activities that maintain healthy bodyweight.

## Introduction

Non-communicable diseases (NCDs), principally cardiovascular disease, cancer and diabetes are the leading cause of death globally and a major contributor to disability [1, 2]. Compelling epidemiological and clinical evidence suggests that overweight and obesity increase the risk of NCDs by over 3-fold compared to those with normal body weight [3, 4]. The prevalence of obesity among young adults in developing countries ranges from 2.3 to 12%, with overweight as high as 28.8% [5]. Obese and overweight people whose weight reduced between early adulthood and midlife halved their risk of dying during the follow-up period [6].

Physical inactivity and dietary habits are the main risk factors contributing to obesity [7–9]. Excess consumption of foods high in energy and low in essential nutrients contributes to overweight or obesity among working-age adults [10]. A physically inactive lifestyle triggers weight gain and vice versa, independent of genetic effects [11]. Lifestyle modifications are still the most widely used and recommended methods to achieve weight reduction, particularly using different dietary strategies while simultaneously undertaking physical activity and exercise [12, 13].

Multiple factors interact in the progress of obesity but gut microbiota play a critical role as bodyweight regulators affecting adiposity and glucose metabolism [14]. Individuals with elevated body mass index (BMI) and adiposity exhibited significantly lower diversity in gut bacteria than those with normal BMI [15]. Physical activity and diet modify gut microbiota composition by increasing microbial diversity [15, 16].

Metabolic biomarker profiles, gut microbiota characteristics and dietary intake of overweight middle-aged and older adults in developing countries are available [17–19] but lack of data exist for overweight Thai young adults. Therefore, this study characterized metabolic and inflammatory biomarkers and gut microbiota profiles of overweight young Thai adults and compared the results to those with normal weight to identify lifestyle factors such as physical activity and dietary patterns within the subject cohort.

## Materials and methods

### Ethics statement

This study was approved by the Ethics Committee of Kasetsart University (License number COA64/068) and registered with the Thai Clinical Trials Registry (TCTR20220204007). Written informed consent was obtained from all participants. The protocol for this trial and supporting CONSORT checklist are available as supporting information; see S1 Checklist and S1 File.

### Study design and subjects

**Fig 1:**
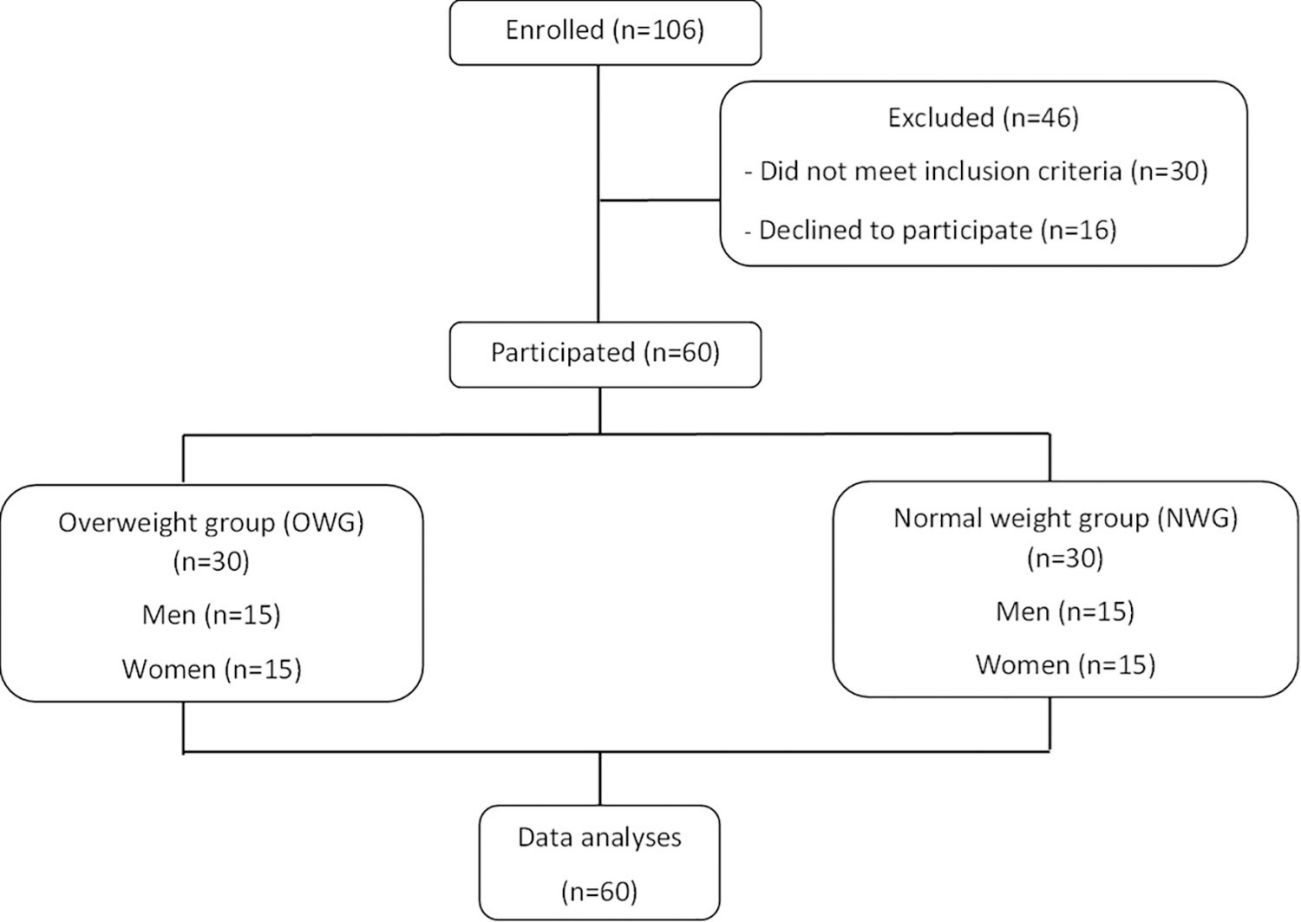
Summary of the recruitment process and study execution.

Sixty subjects aged 20–40 were recruited in March 2022 from Nakhon Pathom Province, Central Thailand via word of mouth, flyers and posters on the Kasetsart University Campus as well as in shops and community buildings in the surrounding area. Email advertisements were sent to Kasetsart University students and staff. Individuals who responded to the advertisements were asked to complete a health and lifestyle questionnaire online or by telephone. Those who fitted the inclusion criteria were invited to the clinical unit for assessment of further inclusion/exclusion criteria (Fig 1). Inclusion criteria were: free from chronic diseases, including cardiovascular disease, diabetes, cancer, and inflammatory or digestive disorders, and had not taken medications, antibiotics or probiotics within three months before participating in the study that might affect lipids, blood clotting or gut microbiota. Exclusion criteria were: asthmatics and consuming more than 21 U/week of alcohol. Subjects who were pregnant and/or breastfeeding were also excluded from the study. The aim of this study was to identify metabolic and inflammatory biomarkers and gut microbiota profiles, and lifestyles of young Thai adults based on their body mass index (BMI). The volunteers were divided into two groups based on the WHO Asian BMI and waist-to-hip ratio (WHR) classification criteria for adults as 30 with normal weight (BMI 18.5–22.9 kg/m^2^, WHR male ≥ 0.95, female ≥ 0.8) and 30 overweight (BMI 23–29.9 kg/m^2^, WHR male < 0.95, female < 0.8). Data of 60 Thai young adults were analyzed for body composition, blood profiles, fecal microbiota, diet habits and physical activity levels.

### Anthropometric measurements

The auxological assessment included height measured by a Harpenden Stadiometer, and weight and body composition assessed via bioelectrical impedance using an Inbody 720 Analyzer. Body composition data of interest comprised total percentage of body fat, body fat mass, and skeletal mass. BMI was computed as weight (in kg) divided by the square of the height in meters [weight/(height)^2^]. Waist circumference (WC) was measured using a Hoechstmass body tape measure at the approximate midpoint between the lower margin of the last palpable rib and the top of the iliac crest. Hip circumference (HC) was measured at the level of greater trochanters, and waist-to-hip ratio (WHR) was calculated as WC (cm) divided by HC (cm).

### Blood pressure measurement

Systolic (SBP) and diastolic blood pressure (DBP) were measured twice with a validated automatic digital blood pressure monitor (OMRON HEM-8712). The average of two readings was used for analysis.

### Analysis of biochemical variables

Blood samples were obtained after overnight fasting lasting at least 10 h. Blood samples obtained via single venepuncture were collected into heparin and EDTA vacutainers (BD). Fasting plasma glucose (FPG) in NaF plasma was measured using the enzymatic (Hexokinase/G-6-PDH) method (Abbott Alinity system, Abbott Park, Illinois, USA). The serum from blood was measured for total cholesterol (TC) using the enzymatic method (Abbott Alinity system, Abbott Park, Illinois, USA), high-density lipoprotein cholesterol (HDL-C) and low-density lipoprotein cholesterol (LDL-C) using accelerator selective detergent method (Abbott Alinity system, Abbott Park, Illinois, USA) and triglycerides (TG) using glycerol phosphate oxidase method (Abbott Alinity system, Abbott Park, Illinois, USA). Insulin (INS) values were analyzed based on a chemiluminescent microparticle immunoassay (CMIA) (Abbott Alinity system, Abbott Park, Illinois, USA). Liver function tests were performed with measurements of serum glutamic oxaloacetic transaminase (SGOT) and serum glutamic pyruvate transaminase (SGPT) from serum without hemolysis by enzymatic NADH (with P-5’-P) method (Abbott Alinity system, Abbott Park, Illinois, USA). Kidney function tests were determined with blood urea nitrogen (BUN) and creatinine (CRE) using the urease and enzymatic method (Abbott Alinity system, Abbott Park, Illinois, USA). C-reactive protein (CRP) was also analyzed as an inflammation marker using turbidimetric/immunoturbidimetric (Abbott Alinity system, Abbott Park, Illinois, USA).

### Lifestyle assessment

Two lifestyle factors were investigated as diet and physical activity. Habitual dietary intakes were collected from study subjects using three-day dietary records (3DDR) and semi-quantitative food frequency questionnaires (FFQ). For 3DDR, each dietary report encompassed an itemized nutritional intake recorded during two weekdays (Monday to Friday) and one weekend. Subjects were shown examples of serving sizes for food and given guidance on their intake recording for each meal. The diet record form consisted of columns to note the meal type/time, type of food, amount, cooking methods, and place. Subjects were required to submit the food record for analysis of mean daily caloric and macronutrient intakes calculated using INMUCAL-Nutrients version 4 software (Institute of Nutrition, Mahidol University, Thailand). For FFQ, subjects were asked to record the frequency of consumption of each food [20]. Physical activity levels were assessed using the International Physical Activity Questionnaire (IPAQ) [21]. The FFQ and IPAQ for this cross-sectional study are available as supporting information; see S2 and S3 Files.

### Fecal sample collection

Fecal samples of all volunteers (approximately 10 g) were collected in a sterilized container, frozen immediately and/or transported with ice packs to the laboratory within 4 h of collection. The samples were stored at -80°C until further use.

### Genomic DNA extraction

Genomic DNA was extracted using the QIAamp DNA Fast Stool Mini Kit (Qiagen, Hilden, Germany) in accordance with protocol Q of the international human microbiome standard (IHMS). Each fecal sample was homogenized and rinsed twice with phosphate-buffered saline (PBS) pH 8 (1:5 w/v). The fecal pellet was then resuspended in 1.5 mL of ASL lysis buffer (Qiagen, Hilden, Germany) and transferred to a 2 mL screw cap tube containing sterile zirconia beads with diameters of 0.1 mm and 1 mm, weighing 0.3 g for each size (BioSpec, Bartlesville, OK, USA). Physical disruption of microbial cells was performed by running a FastPrep-24 benchtop instrument (MP Biomedicals, Santa Ana, CA, USA) at maximum speed for 8 minutes and 30 seconds, with a series of 5 minutes resting on the ice for every minute of beating. The purity and quantity of DNA were determined using a Nanodrop 2000c (Thermo Scientific, Waltham, MA, USA). The genomic DNA was stored at -20°C until further analysis.

### Fecal microbiome analysis

Fecal microbiota are conventionally analyzed using 16S rRNA gene sequencing data. The genomic DNA of each sample was sent for sequencing to the outsource company (Novogene Co., Ltd., Beijing, China). The sequencing process was carried out by amplifying the V3-V4 region of the 16s rRNA gene using forward primer 341F (CCTAYGGGRBGCASCAG) and reverse primer 806R (GGACTACNNGGGTATCTAAT). The barcode sequences were integrated with a forward primer at the 5’ end. Amplification was performed by using Phusion® High-Fidelity PCR Master Mix (New England Biolabs, Ipswich, MA, USA) under the following conditions: Initial denaturation at 95°C for 2 min; 35 amplification cycles of 95°C for 30 s, 57°C for 30 s, 72°C for 30 s and a post-extension at 72°C for 10 min. The PCR product was mixed with 1X loading buffer containing SYBR Green dye and run in 2% agarose, 80 V, 40 min. Samples with a bright band between 400 and 450 bp were mixed at an equidensity ratio and purified with Qiagen Gel Extraction Kit (Qiagen, Hilden, Germany). Sequencing libraries were prepared using NEBNext® Ultra™ DNA Library Prep Kit for Illumina (New England Biolabs, Ipswich, MA, USA) according to the manufacturer’s recommendation. The library quality was assessed on a Qubit 2.0 Fluorometer (Thermo Scientific, Waltham, MA, USA) and Agilent Bioanalyzer 2100 system (Agilent Technologies, Santa Clara, CA, USA). The library was then sequenced on Illumina NovaSeq 6000 (Illumina, San Diego, CA, USA).

### Bioinformatics

Paired-end reads were assigned to each sample based on the previously linked barcode sequence. Reads were merged into a single sequence and the primers were trimmed using the search_pcr2 command in USEARCH v11.0.667 [22]. Merged reads shorter than 300 bp or having expected errors higher than 1.0 were discarded. The remaining quality-filtered reads were corrected for sequence errors by implementing the UNOISE algorithm [23]. An amplicon sequence variances (ASVs) table was produced with USEARCH and then mapped with the representative sequence to obtain the abundance. Taxonomy was assigned using the SINTAX algorithm [24] and Ribosomal Database Project (RDP) training set v18 database [25] with an 80% identity cut-off. Microbial diversity metrics were calculated from the ASVs table with USEARCH. The alpha diversity was determined by calculating the Shannon, Chao1, and Pielou’s index, whereas the beta diversity metric was calculated based on the Bray-Curtis dissimilarity.

### Sample size

A mean reduction in HDL-C was chosen as a clinically significant end point, since it has been strongly associated with and largely attributable to obesity [26, 27]. The power calculation was based on a known mean overweight Thai adult HDL-C of 42.57 mg/dL and standard deviation of 6.37mg/dL [17]. A sample of 60 participants would have at least 80% power at 5% level of significance (two-sided) to detect a difference in HDL-C between the normal and overweight Thai adults.

### Statistical analysis

Statistical analyses were performed using SPSS® (version 26.0; SPSS Inc., Armonk, NY, USA). All statistical tests were a 5% significance level maintained throughout the analyses. Variables were summarized as mean and standard deviation (mean ± SD). Distribution of the data for each variable was evaluated using the Kolmogorov-Smirnov test. Some response variables were log-transformed with base 10 to approximate normality in case of not normally distributed data. The independent samples t-test was used to determine significant differences between the means of the two independent groups. Proportions were compared using the Chi-square test, with p-value < 0.05 considered significant. For bioinformatic data, all statistical analyses and visualizations were carried out in XLSTAT 2019.2.2, PRIMER 7 v7.0.20, and GraphPad Prism 9.0.0. Non-parametric statistical analysis was selected based on the normality of data distribution assessed by the Shapiro-Wilk. All statistical tests maintained a 5% significance level throughout the analyses. The significance of the taxon was calculated using the Kruskal-Wallis test and Dunn’s post hoc analysis at the 95% confidence level [28]. Adjustment for multiple comparison analyses was performed by employing the FDR algorithm [29]. The raw 16s amplicon sequences used in this study have been deposited at the NCBI shorts read archive (SRA) with the Bio Project accession number PRJNA877411.

## Results

### Subject demographics

Table 1 shows the characteristics of the study subjects. The 60 subjects had a mean age of 30 ± 9.00 years, with 30 in the overweight group (OWG) and 30 in the normal weight group (NWG). Both study groups had an equal ratio of males and females. Individuals in OWG had significantly higher BMI, WHR, BFM, % BF, SM, SBP, TG, CRP and INS than those in NWG, while HDL-C and BUN levels were lower in NWG compared to OWG (p<0.05).

**Table 1:** Baseline characteristics of study participants.

Characteristic	Total (n = 60)	NWG (n = 30)	OWG (n = 30)	p-value
Age (year)	30± 9.00	27 ± 7.50	33 ± 9.48	0.008*
Gender (male/female)	30/30	15/15	15/15	1.000
Height (cm)	167.00 ± 8.36	167.20 ± 8.12	166.80 ± 8.72	0.891
Weight (kg)	68.78 ± 13.75	58.88 ± 5.76	78.68 ± 12.20	< 0.001*
BMI (kg/m^2^)	24.68 ± 4.65	21.08 ± 1.38	28.28 ± 3.92	< 0.001*
SBP (mmHg)	119 ± 12	114 ± 13	124 ± 10	0.003*
DBP (mmHg)	77 ± 10	75 ± 8	79 ± 10	0.074
BFM (kg)	20.29 ± 9.93	13.00 ± 4.96	27.57 ± 8.14	< 0.001*
% BF	28.30 ± 10.49	21.94 ± 8.41	34.66 ± 8.32	< 0.001*
SM (kg)	27.08 ± 5.77	25.51 ± 4.55	28.64 ± 6.47	0.034*
TC (mg/dL)	198.67 ± 37.23	200.90 ± 32.80	196.43 ± 41.64	0.646
LDL-C (mg/dL)	132.69 ± 37.39	131.12 ± 32.06	134.26 ± 42.55	0.748
HDL-C (mg/dL)	52.89 ± 12.59	58.63 ± 10.57	47.33 ± 12.04	< 0.001*
TC (mg/dL)	102.42 ± 65.00	82.43 ± 60.98	122.40 ± 63.65	0.016*
FBG (mg/dL)	85.40 ± 7.12	85.87 ± 6.11	84.93 ± 8.08	0.616
CRP (mg/dL)	2.18 ± 3.57	0.69 ± 0.78	3.67 ± 4.55	< 0.001*
BUN(mg/dL)	12.55 ± 3.30	13.40 ± 3.49	11.70 ± 2.91	0.045*
CRE (mg/dL)	0.81 ± 0.18	0.85 ± 0.18	0.78 ± 0.18	0.126
INS (μU/mL)	9.23 ± 5.55	6.68 ± 2.42	11.77 ± 6.58	< 0.001*
SGOT (U/mL)	24.25 ± 23.69	0.22 ± 0.04	0.22 ± 0.37	0.665
SGPT (U/mL)	25.05 ± 20.13	22.00 ± 17.58	28.10 ± 22.27	1.000

Data are presented as mean ± standard deviation or n (%).

*p-value for differences between the overweight group (OWG) and normal weight group (NWG) by t-test for continuous variables (p<0.05). Abbreviations: SBP, systolic blood pressure; DBP, diastolic blood pressure; BFM, body fat mass; BF, body fat; SM, skeletal muscle mass; TC, total cholesterol; LDL-C, low density lipoprotein cholesterol; HDL-C, high density lipoprotein cholesterol; TC, triglyceride; FBG, fasting blood glucose; CRP, c-reactive protein; BUN, blood urea nitrogen; CRE, Creatinine; INS, Insulin; SGOT, serum glutamic oxaloacetic transaminase; SGPT, serum glutamate pyruvate transaminase.

### Composition and fecal microbiota diversity

**Fig 2:**
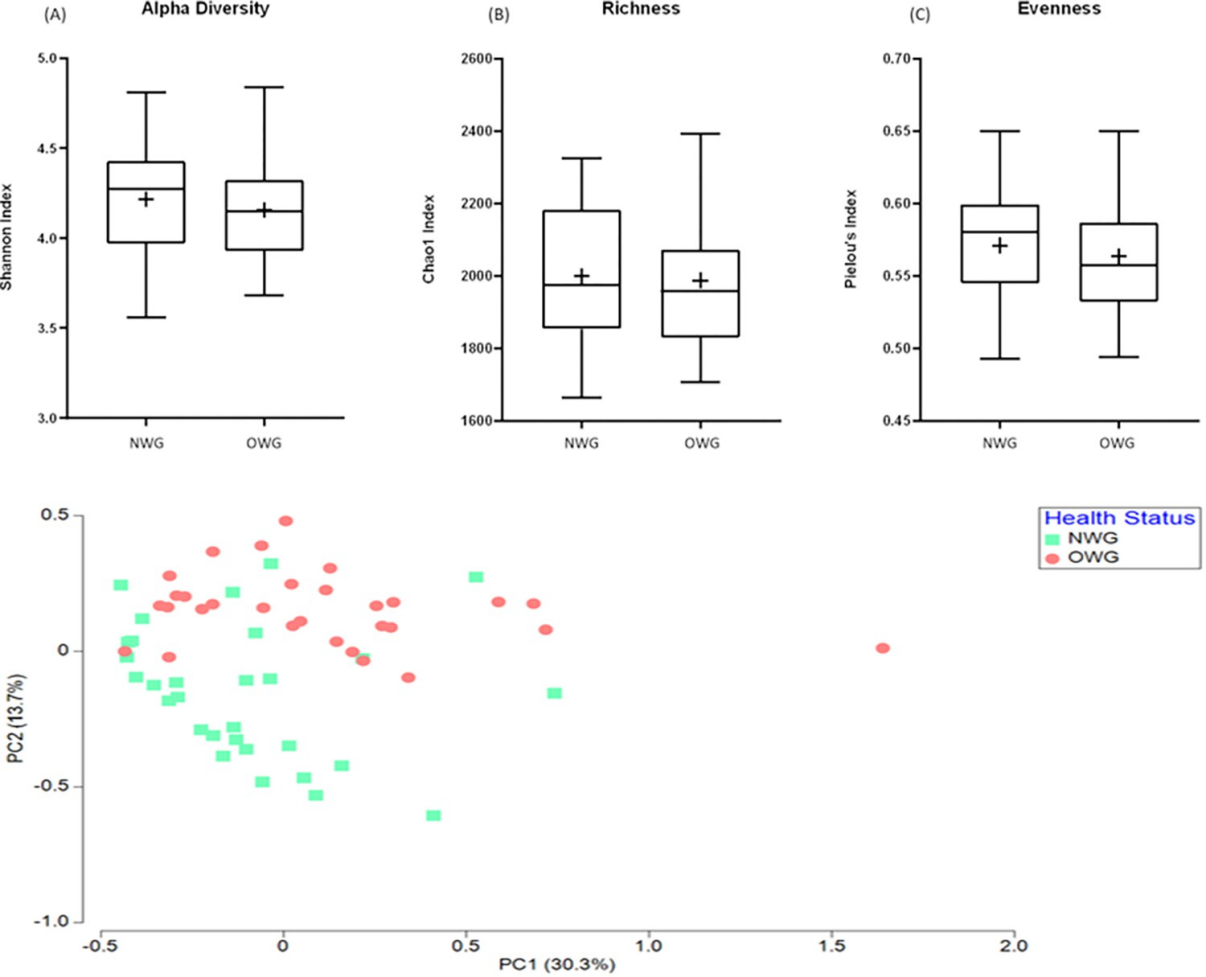
Diversity of fecal microbiota in OWG and NWG. **(A)** Alpha diversity index between groups. **(B)** Beta-diversity visualized by PCA analysis. Each point represents one sample and points with the same color are in one group.

As shown in Fig 2A, differences in alpha diversity (Shannon index), richness (Chao1 index) and evenness (Pielou’s index) between OWG and NWG were not significant. Furthermore, PCA showed that samples in the two groups were clustered into two different areas (Fig 2B).

**Fig 3:**
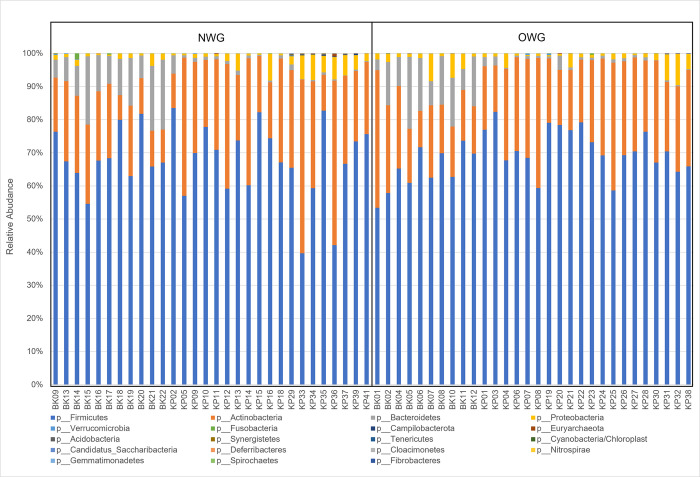
Fecal microbiota composition in OWG and NWG. Bar plot of fecal microbiota composition at the phylum level.

Fecal microbiota of all samples were classified into 281 genera, 125 families, 36 classes, 19 phyla and 2 kingdoms. At the phylum level, there were no differences between OWG and NWG in fecal microbiota composition. The four predominant phyla in both groups were *Firmicutes*, *Actinobacteria*, *Bacteroidetes and Proteobacteria*. There were no significant differences in the ratio of *Firmicutes/Bacteriodetes* (F/B) between the two groups (Fig 3). At the family level, relative abundances of *Clostridiaceae-1*, *Bacillaceae-1* and *Wohlfahrtiimonas* in OWG were significantly higher than in NWG, while relative abundances of *Eggerthellaceae*, *Rikenellaceae*, *Nocardioidaceae* and *Chitinophagaceae* were significantly lower than in NWG (p<0.05). At the genus level, relative abundances of *Clostridium sensu stricto*, *Senegalimassilia*, *Enterobacter*, *Citrobacter*, *Bacillus*, *Paraclostridium* and *Lancefieldella* were significantly higher in OWG than in NWG, while the relative abundances of *Alistipes*, *Fecalicatena*, *Oscillibacter*, *Limosilactobacillus*, *Slackia*, *Ruthenibacterium*, *Gordonibacter* and *Longibaculum* were significantly higher in NWG than in OWG (p<0.05).

### Dietary intake and eating habits

**Fig 4:**
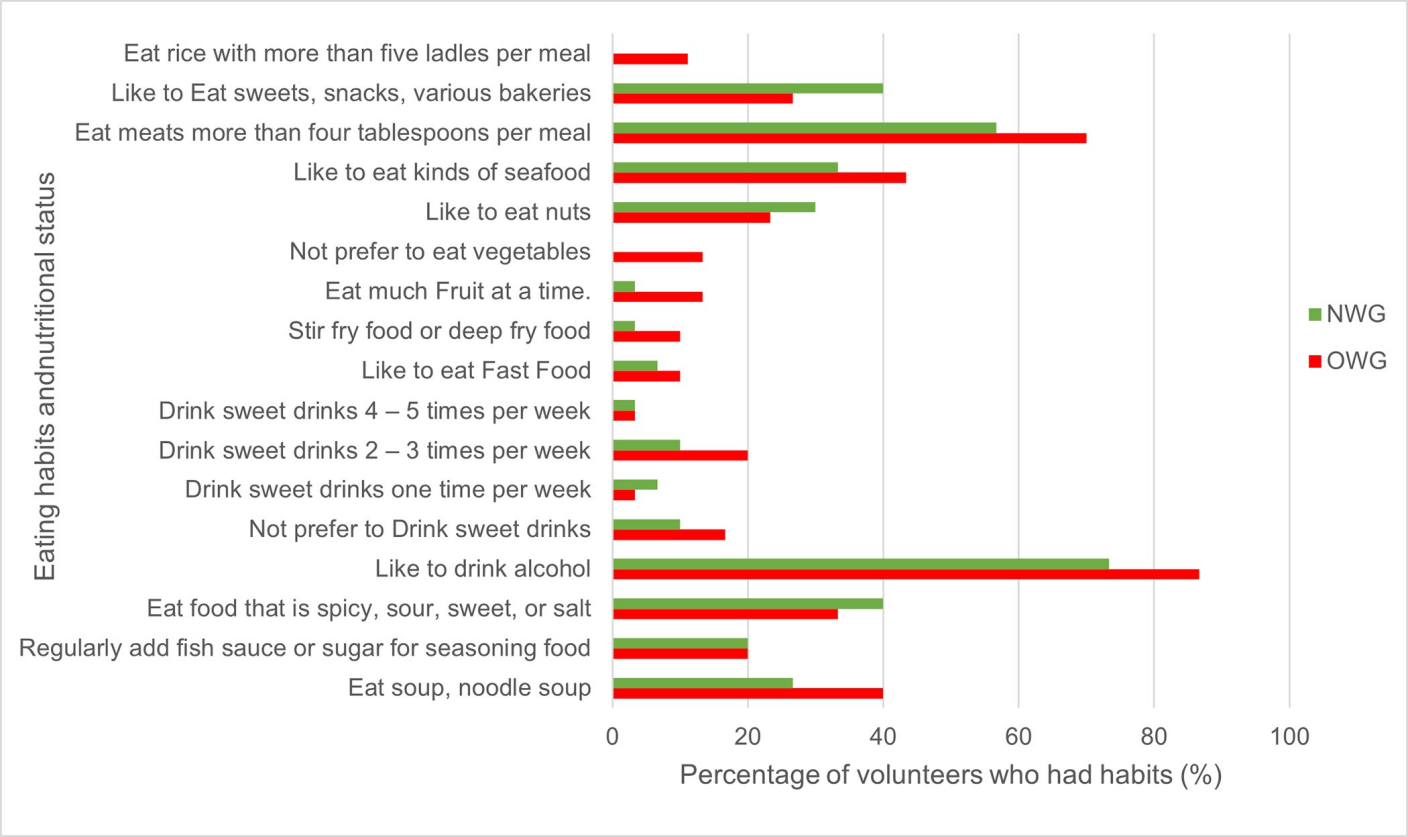
Comparison of eating habits and nutritional status of subjects.

Nutrient intakes assessed by 3DDR are shown in Table 2. There were no significant differences in daily energy among the two groups; however, mean values of several nutrient intakes showed significant differences between OWG and NWG. Protein-vegetable and iron-vegetable intakes were significantly higher for OWG than NWG. A proportion of the study participants conformed to Thai RDIs for energy and nutrient intakes, as shown in Table 2. The OWG adhered more to energy, carbohydrate, fat and protein recommendations than NWG [30]. Fig 4 compares eating habits between OWG and NWG from FFQ. Individuals in OWG reported greater portion size of food intake and higher consumption of sweet fruits, fast food, food high in fat such as coconut curry, deep-fried food, stir-fried food, food high in carbohydrates such as rice, soup high in salt, processed meat, beverages and alcohol than NWG, while NWG consumed more nuts, grain, vegetables, sweets, snacks, bakeries and spicy food.

**Table 2:** Energy and nutrient intake of subjects assessed with 3DDR.

Nutrition	NWG (n = 30)	%RDI	OWG (n = 30)	%RDI	p-value
Energy (kcal)	1,349.50 ± 405.90	66.8%	1,425.21 ± 633.49	71.8%	0.584
Carbohydrate (g)	149.99 ± 48.01	37.6%	172.21 ± 69.32	44.0%	0.154
FAT (g)	54.54 ± 23.33	109.1%	51.51 ± 32.51	103.0%	0.680
Protein (g)	64.66 ± 26.69	114.4%	68.19 ± 40.84	121.8%	0.693
Protein-animal (g)	47.29 ± 25.91	NA	48.18 ± 36.67	NA	0.914
Protein-vegetable (g)	8.51 ± 3.55	NA	12.06 ± 6.13	NA	0.008*
Calcium (mg)	321.57 ± 158.81	40.2%	345.89 ± 260.99	43.2%	0.664
Iron (mg)	8.10 ± 3.82	51.4%	8.84 ± 4.18	56.1%	0.476
Iron-animal (mg)	4.11 ± 2.33	NA	3.97 ± 3.43	NA	0.851
Iron-vegetable (mg)	2.69 ± 1.38	NA	3.43 ± 1.31	NA	0.037*
Vitamin A (RAE)	567.63 ± 987.58	81.1%	501.83 ± 982.21	71.7%	0.797
Thiamin (mg)	0.82 ± 0.50	71.3%	1.73 ± 3.47	150.4%	0.158
Riboflavin (mg)	0.90 ± 0.35	75.0%	0.95 ± 0.52	79.2%	0.712
Vitamin C (mg)	49.16 ± 94.98	53.1%	59.94 ± 84.61	64.8%	0.644
Niacin (mg)	9.00 ± 5.39	60.0%	11.08 ± 5.23	73.9%	0.134
Ash (mg)	34.83 ± 19.85	NA	54.01 ± 70.47	NA	0.157
Beta-carotene (mcg)	1727.41 ± 2076.85	NA	1643.24 ± 1576.28	NA	0.861
Copper (mg)	1.42 ± 1.10	97.9%	1.93 ± 1.16	133.1%	0.086
Crude fiber (g)	0.55 ± 0.75	NA	0.80 ± 1.54	NA	0.491
Dietary fiber (g)	18.55 ± 10.64	74.2%	23.42 ± 12.72	93.7%	0.113
Cholesterol (mg)	1030.29 ± 550.57	NA	863.74 ± 539.76	NA	0.242
Potassium (mg)	3201.82 ± 1823.37	84.3%	3423.17 ± 1669.07	91.3%	0.626
Magnesium (mg)	128.58 ± 115.13	45.1%	138.19 ± 84.82	47.7%	0.714
Sodium (mg)	6959.63 ± 4562.20	520.1%	10484.51 ± 16177.80	791.3%	0.255
Phosphorus (mg)	1703.54 ± 743.72	243.4%	1794.49 ± 835.74	256.4%	0.658
Phytate (mg)	40.24 ± 60.73	NA	24.74 ± 38.65	NA	0.251
Retinol (mcg)	1292.12 ± 2620.02	NA	1334.39 ± 2959.72	NA	0.953
Total saturated fatty acid (g)	43.72 ± 26.14	198.7%	44.02 ± 28.34	200.1%	0.966
Selenium (mcg)	102.44 ± 54.40	186.3%	100.92 ± 54.60	183.5%	0.915
Sugar (g)	124.49 ± 73.78	NA	143.65 ± 103.72	NA	0.413
Vitamin B12 (mcg)	4.31 ± 4.11	179.6%	2.87 ± 2.80	119.6%	0.127
Vitamin B6 (mg)	12.1 ± 0.71	930.8%	1.14 ± 0.71	87.7%	0.712
Vitamin E (mg)	70.65 ± 293.45	588.8%	156.28 ± 501.32	1302.3%	0.423
Water (g)	2467.68 ± 1763.80	NA	2976.60 ± 1746.13	NA	0.266
Zinc (mg)	11.10 ± 6.17	106.7%	13.51 ± 5.85	134.4%	0.125

Data are presented as mean ± standard deviation.

*p-value for difference between the overweight group (OWG) and the normal weight group (NWG) (p< 0.05). Abbreviations: RDI, recommended dietary intake; NA, not applicable.

### Physical activity and sedentary behavior

**Fig 5:**
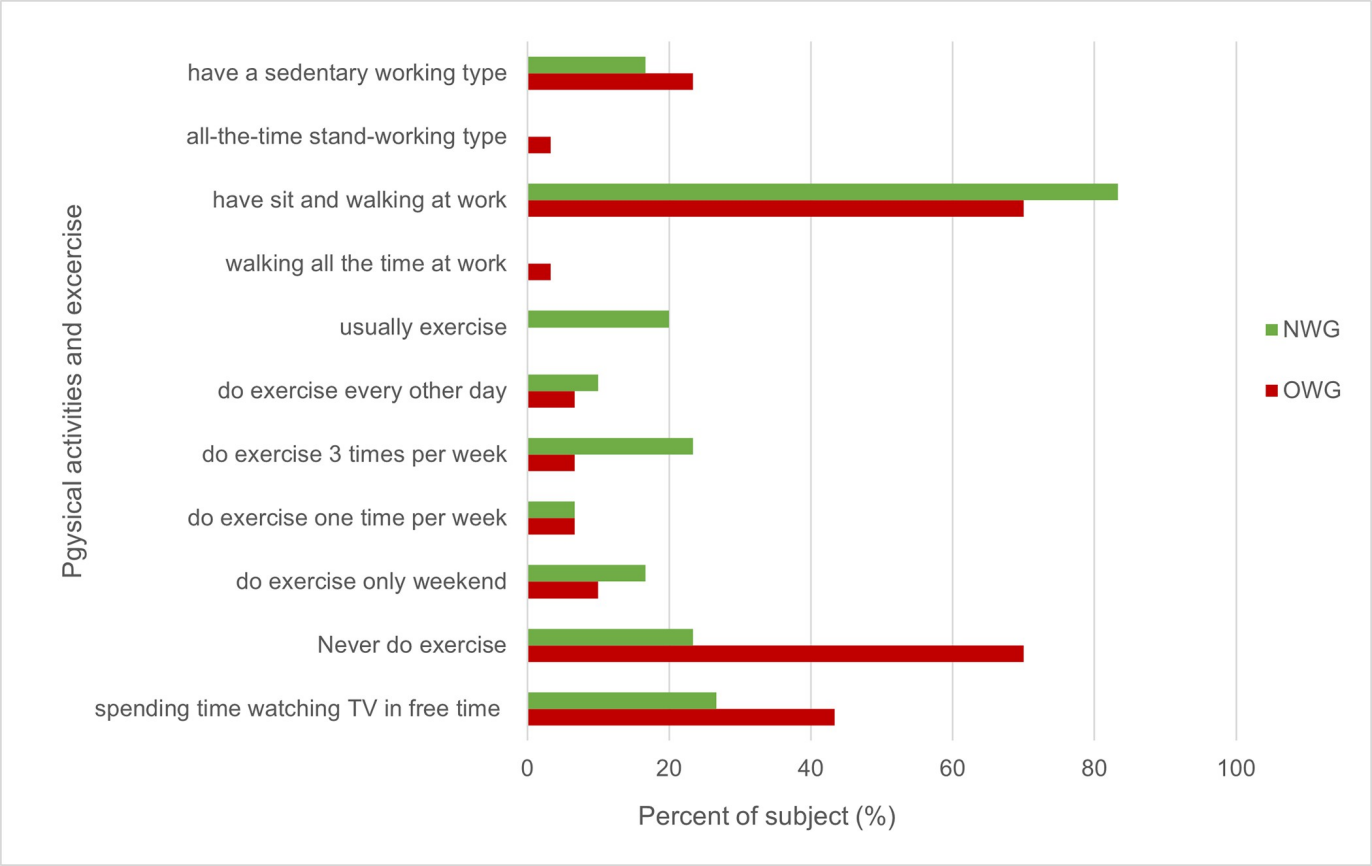
Comparison of physical activity and sedentary behavior between NWG and OWG.

The time spent sitting and physical activity level in the study subjects varied significantly between OWG and NWG (p<0.05). OWG spent more time sitting, watching television and lying down with lower physical activity levels than NWG (Fig 5).

## Discussion

Excessive body weight is a well-known risk factor for non-communicable diseases (NCDs) [31]. In this study, we investigated the differences in metabolic profiles, gut microbiota and lifestyle factors in overweight (OWG) and normal weight (NWG) young Thai adults. These comparisons offer a clear picture of the reasons for overweight young adults in developing countries. In our clinical setting, we grouped the subjects by BMI and waist circumference (WC) following Asia criteria to screen for abdominal obesity because BMI alone does not account for body fat distribution [32]. We found that the mean age of OWG was higher than NWG (p≤0.05); however, all subjects in both groups were healthy young adults. Evidence shows that Thai people increase their BMI when age increases [33]. The main finding of this study was the statistically significant difference in some metabolic and inflammatory profiles and fecal microbiota composition at the genus and family levels, as well as dietary habits, physical activity and sedentary behavior in OWG compared with NWG. Results showed significantly high levels of TAG, INS, CRP and SBP, with low levels of HDL-C and BUN in OWG, concurring with previous studies [3, 26, 34, 35]. Average clinical blood values were in the normal reference range in OWG because the samples were free from chronic diseases. Obesity is associated with abnormal lipid metabolism and a higher risk of NCDs in adults [36]. Elevated levels of TAG and SBP and low levels of HDL-C are high risk factors for coronary heart disease [37]. Overweight individuals (BMI 25–29.9 kg/m^2^) had a 60% higher risk of CVD mortality [38]. Each 1-mg/dL elevation in HDL-C reduced CVD risk by 2% to 3% [39]. For SBP, each 5 mm Hg increment resulted in a 4% higher risk of cardiovascular events [40]. High FI level is associated with high rates of weight gain [41] as an accurate predictor of insulin resistance (IR) [42], which is a fundamental aspect of the etiology of type 2 diabetes [43]. In a large cohort of 10,745 subjects, low-grade systemic inflammation and CRP increased as BMI increased [44]. Levels of CRP at more than 1.0 mg/dL have been associated with a 2- to 3-fold increase in risk of NCDs in healthy men and women [45]. Our results indicated that individuals in OWG had average CRP concentration of 3.67 ± 4.55 mg/dL with increased risk of NCDs.

Lifestyles, such as dietary habits and physical activity, play an important role in obesity development [46]. Nutrition transition in developing countries leads to dietary intakes of micronutrient-poor, energy-dense foods as important determinants of overweight/obesity [47]. In our study, only a few dietary factors were significantly different between OWG and NWG. The absolute amount of dietary fat and protein intake from 3DDR was higher in OWG and exceeded the current Thai recommendation. Many dietary habits contribute to obesity including eating frequency, meat consumption and calories from protein and snack consumption [36]. We observed more unhealthy eating habits such as large portion size per meal, high consumption of sweet fruits, food rich in fat and carbohydrates, processed meat, beverages and alcohol and consuming fewer vegetables, nuts, grain and spices in the OWG. These increased clinical biomarkers contributed to NCDs in overweight young adults.

We found that fiber intake was higher in the OWG compared to NWG. The OWG had careers in agriculture and farming with low incomes, preferring to find plants, vegetables and insects that are available from natural sources for consumption, resulting in fiber and plant-based protein and more animals than the NWG, who mostly worked in offices. Office workers in Thailand often consume high-fat meat that is grilled or deep fried and may result in the intake of fat and cholesterol from food. The NWG consumed snacks and bakery products that contained more fat and/or cholesterol than the OWG. Data on the amount of vitamin E that Thai people have is limited. This study showed that both the NWG group and the OWG group received sufficient vitamin E from diet. Most Thai dishes are cooked by stir-frying or deep fried, while coconut milk curry contains oil. The most popular cooking oils are rice bran oil, soybean oil and palm oil which is a source of vitamin E. However, vitamin E intakes in both groups were within the range set by the European Food Safety Authority (EFSA) [48]. The study also has some limitations. 3DDR has a relatively high respondent burden that may affect foods or quantities that are selected. Subjects also tend to record less diligently as the duration increases, while FFQ relies on the ability of long-term recall [49]. Furthermore, our study was conducted during the COVID-19 pandemic and this may have affected eating behaviors [50].

Physical activity plays a fundamental role in balancing energy and weight, NCD risk factors and promoting general well-being. According to the WHO recommendations, an adult needs to undertake moderate-intensity physical activity for at least 150 minutes throughout the week [51]. We found that individuals with normal BMI adhered to WHO physical activity recommendations. Elevated BMI is considerably reduced by higher physical activity levels. The Rotterdam Study also mentioned that overweight participants with low levels of physical activity had a 1.33 and 1.35 times higher risk of CVD than the normal weight participants with high levels of physical activity [52]. Lifestyle modifications involving specific changes in diet, physical activity and exercise are considered cornerstones of obesity management.

Several studies have shown direct associations between sedentary behavior (SB) such as sleeping, sitting, lying down and watching television and increased risk of NCDs [53, 54]. In line with these, we also found that OWG spent more time each day sitting and watching TV than NWG and less time doing exercise. Experimental studies in humans have demonstrated that an increase in SB is associated with reduced energy expenditure, development of IR and accumulation of abdominal fat [55]. The association between SB and adiposity was partially explained by food and beverage consumption during TV viewing but not by a reduction in overall leisure-time physical activity [56]. Our results suggested that overweight individuals spend more time sitting and are physically less active because of their body weight. Current key recommendations encourage people to increase their physical activity levels by adopting a healthy diet corresponding to energy expenditure, while also reducing time spent sitting down and watching TV, to prevent accumulation of excess body weight [57].

The gut microbiota has recently been recognized as a key environmental factor driving metabolic diseases [58, 59]. Dysbiosis in intestinal microbiota has been associated with obesity [60, 61]. Previous studies showed that the structure, function and diversity of the gut microbiota among people with obesity are different from those with normal weight [62, 63]. Our study found no significant difference in fecal microbiota diversity and composition at the phylum level, similar to Gruneck et al. [17]. Research indicated that the amount and structure of gut microflora are 57% influenced by dietary habits and 12% by genetic factors [64]. In our study, no significant differences in daily energy were found among the two groups, contributing to the similarity of fecal microbiota diversity and composition at the phylum level in both groups. The four predominant phyla in both groups were *Firmicutes*, *Actinobacteria*, *Bacteroidetes* and *Proteobacteria*, concurring with previous studies that reported these phyla as contributing the majority of human gut bacteria [65]. Foods that strongly affect gut microflora include resistant starch, fats, fructose, glucose, casein and arginine[66]. Consuming starchy foods such as rice, noodles and white flour resulted in a large proportion of *Firmicutes* and *Actinobacteria* [67, 68]. However, at the genus and family level, significant differences in bacterial compositions were observed between NWG and OWG (p<0.05). Relative abundances of *Alistipes*, *Fecalicatena*, *Oscillibacter*, *Limosilactobacillus*, *Slackia*, *Ruthenibacterium*, *Gordonibacter* and *Longibaculum* were significantly higher in NWG than in OWG. *Oscillibacter* has also been suggested to promote human leanness and was enriched in healthy-weight subjects [69]. *Citrobacter*, a common urinary pathogen, was higher in NWG and related to the higher value of BUN in our subjects [70, 71] but opposite findings have also been reported [72, 73]. Gut microbiota reduce the development of obesity by regulating energy and improving the ability to ferment and digest polysaccharide foods which produce short chain fatty acids that stimulate lipogenesis, causing the accumulation of triglyceride fat in fat cells [74]. Research has shown that succinate, produced from microorganisms in the phylum *Bacteroidetes* (Prevotella), can stimulate gluconeogenesis, resulting in fat accumulation and increased body weight [75]. Furthermore, changes in intestinal microbiota suggest that physical activity could increase microbial variance, thereby neutralizing obesity progression and diminishing body weight [76].

## Conclusions

Findings from this cohort study showed that being overweight disturbs the lipid and glucose metabolisms in otherwise healthy Thai young adults. Unhealthy lifestyles, including lack of sufficient physical activity and unhealthy dietary habits, might contribute to the development of overweight. Our results offer supportive evidence for public health policies and healthcare professionals to encourage people to modify their lifestyles to reduce the risk of being overweight. However, further studies are needed with larger sample sizes to identify the correlation between fecal microbiota-derived metabolites and health status in overweight young adults.

## Supporting information

S1 Checklist CONSORT checklist. (PDF)

S1 File Trial protocol. (DOCX)

S2 File Food Frequency Questionnaire (FFQ). (DOCX)

S3 File (DOCX)
