# Supplementary material for: Metabolic and inflammatory profiles, gut microbiota and lifestyle factors in overweight and normal weight young thai adults
Source: PLoS One. 2023 Jul 14;18(7):e0288286. doi: 10.1371/journal.pone.0288286 (PMC10348517; doi:10.1371/journal.pone.0288286)
Supplement: S1 File — (DOCX) [file pone.0288286.s002.docx]

**Protocol S1 – Trial protocol**

***Trial registration:*** *TCTR20220204007 (Australian New Zealand Clinical Trials Registry).*

The study on metabolic and inflammatory biomarkers and gut Microbiota in overweight Thai young adults are scarce. The principal aim of this study will characterize metabolic and inflammatory biomarkers and gut microbiota profiles of overweight young adults of Thai origin and compared the results to those with normal weight to identify lifestyle factors as physical activity and dietary patterns within the subject cohort.

**Inclusion criteria**

1. Overweight (BMI more than or equal to 23 kg/m^2^) Age 20 years to 50 years
2. Normal weight ((BMI 18.5 to 22.9 kg/m^2^) Age 20 years to 50 years

**Exclusion Criteria**

1. Any chronic diseases
2. Medication for dyslipidemia, type 2 diabetes or elevated blood pressure
3. History of intestinal surgery
4. Pregnancy/breastfeeding
5. The use of antibiotics, pro-prebiotics within 3 months

**Study Design**


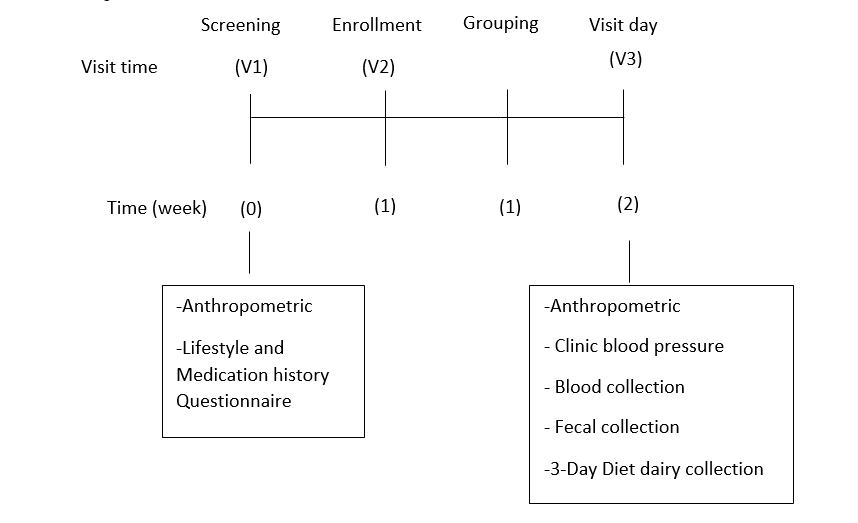


Overweight (n=60) and Normal weight (n=60)

**Anthropometric measurements**

- Height will be measured by a Harpenden Stadiometer
- Weight and body composition (total percentage of body fat, body fat mass and skeletal mass) will be assessed via bioelectrical impedance using an Inbody 720 Analyzer. Body composition data of interest comprised.
- BMI will be computed as weight (in kg) divided by the square of the height in meters [weight/(height)^2^].
- Waist circumference (WC) will be measured using a Hoechstmass body tape measure at the approximate midpoint between the lower margin of the last palpable rib and the top of the iliac crest.
- Hip circumference (HC) will be measured at the level of greater trochanters, waist-to-hip ratio (WHR) will be calculated as WC (cm) divided by HC (cm) and waist-to-height ratio (WHtR) will be calculated as WC (cm) divided by height (cm).

**Blood pressure measurement**

- Systolic (SBP) and diastolic blood pressure (DBP) will be measured twice with a validated automatic digital blood pressure monitor (OMRON HEM-8712). The average of two readings will be used for analysis.

**Analysis of biochemical variables**

- Blood samples will be obtained after overnight fasting (10–12 h) with minimum stasis to avoid the influence of postprandial lipid increase on hemorheological parameters.
- Blood samples obtained via single venepuncture will be collected into heparin and EDTA vacutainers (BD).
- Fasting plasma glucose (FPG), total cholesterol (TC), high-density lipoprotein cholesterol (HDL-C), low-density lipoprotein cholesterol (LDL-C) and triglycerides (TG) will be measured by enzymatic kit methods.
- Insulin values will be analyzed based on a chemiluminescent microparticle immunoassay (CMIA).
- Liver function tests will be performed with measurements of serum glutamic oxaloacetic transaminase (SGOT) and serum glutamic pyruvate transaminase (SGPT). Kidney function tests will be determined with blood urea nitrogen (BUN) and creatinine.
- C-reactive protein (CRP) will be analyzed as an inflammation marker using turbidimetric/Immunoturbidimetric.

**Lifestyle assessment**

- Lifestyle factors will be recorded in an itemized food diary as frequency of intake and physical activity recall.
- Habitual dietary intakes will be collected by a nutritionist using three-day dietary records (3DDR) and semi-quantitative food frequency questionnaires (FFQ).
- For 3DDR, each dietary report encompassed an itemized nutritional intake recorded during two weekdays (Monday to Friday) and one weekend. Subjects will be shown examples of serving sizes for food and given guidance on their intake recording for each meal. The diet record form consisted of columns to note the meal type/time, type of food, amount, cooking methods and place. Subjects will be required to submit the food record for analysis of mean daily caloric and macronutrient intakes calculated using INMUCAL-Nutrients version 4 software (Institute of Nutrition, Mahidol University, Thailand).
- For FFQ, subjects will be asked to record the frequency of consumption of each food during a specified period. Physical activity levels will be assessed using the International Physical Activity Questionnaire (IPAQ) [20].

**Fecal sample collection**

- Fecal samples of all volunteers (approximately 10 g) will be collected in a sterilized container, frozen immediately and/or transported with ice packs to the laboratory within 4 h of collection.
- The samples will be stored at -80 °C until further use.

**Genomic DNA extraction**

- Genomic DNA will be extracted using the QIAamp DNA Fast Stool Mini Kit (Qiagen, Hilden, Germany) in accordance with protocol Q of the international human microbiome standard (IHMS).

**Gut microbiome analysis**

- Gut microbiota are conventionally analyzed using 16S rRNA gene sequencing data. The genomic DNA of each sample will be sent for sequencing to the outsource company (Novogene Co., Ltd., Beijing, China).

**Bioinformatics**

Paired-end reads will be assigned to each sample based on the previously linked barcode sequence. Reads will be merged into a single sequence and the primers will be trimmed using the search_pcr2 command in USEARCH v11.0.667

**Statistical analysis**

Statistical analyses will be performed using SPSS® (version 26.0; SPSS Inc., Armonk, NY, USA). Variables will be summarized as mean and standard deviation (mean ± SD) and the independent samples t-test will be used to determine significant differences between the means of two independent groups. Proportions will be compared using the Chi-square test, with p-value < 0.05 considered significant. For bioinformatic data, all statistical analyses and visualizations will be carried out in XLSTAT 2019.2.2, PRIMER 7 v7.0.20, and GraphPad Prism 9.0.0. Non-parametric statistical analysis will be selected based on the normality of data distribution assessed by the Shapiro-Wilk algorithm. The significance of the taxon will be calculated using the Kruskal-Wallis test and Dunn’s post hoc analysis at the 95% confidence level. Adjustment for multiple comparison analyses will be performed by employing the FDR algorithm. The raw 16s amplicon sequences used in this study have been deposited at the NCBI shorts read archive (SRA) with the Bio Project accession number PRJNA877411.
