# Supplementary material for: Metabolic and inflammatory profiles, gut microbiota and lifestyle factors in overweight and normal weight young thai adults
Source: PLoS One. 2023 Jul 14;18(7):e0288286. doi: 10.1371/journal.pone.0288286 (PMC10348517; doi:10.1371/journal.pone.0288286)
Supplement: S2 File — (DOCX) [file pone.0288286.s003.docx]

Medical and Food Frequency Questionnaire

DD/MM/YY .....................................................

Part 1 Personal details

1. ID................................................................................................... Gender 🗆 male 🗆 female
2. Body weight ..............................kg Height.............................. cm BMI .......................kg/m^2^

Waist size...................cm (.......................inch) Hip size .....................cm (........................inch)

Blood pressure.......................... mmHg Heart rate .......................... beats/minutes

1. Address House number ................................... Soi/Village........................................... Street........................................... Sub-district..........................................District...............................................

Province............................................. postcode.......................................

1. House number ................................................. Telephone number......................................... Office number...................................................
2. DD/ MM / YY Birth............................................................ Age .................................
3. Ethnicity 🗆 Thai 🗆 Chinese 🗆 other.................................................................
4. Religion 🗆 Buddhism 🗆 Christianity 🗆 Islam 🗆 other...................................
5. Marital status 🗆 single 🗆 married 🗆 widowed 🗆 divorced 🗆 separated

🗆 other.................................

1. Do you have any children? 🗆 Yes .....................(number) 🗆 No
2. Education

🗆 unlettered 🗆 Primary school 🗆 Secondary school

🗆 Diploma or Associate’s degree

🗆 Bachelor’s degree or higher 🗆 other.......................................................

1. occupation

🗆 unemployed 🗆 government or state enterprise employee

🗆 company employee 🗆 own business 🗆 other .............................

1. Your income per month

🗆 Lower 5,000 Baht 🗆 5,000 – 10,000 Baht 🗆 10,001 – 20,000 Baht

🗆 20,001 – 30,000 Baht 🗆 Upper 30,000 Baht

1. Resources for information about this training program

🗆 Radio 🗆 Poster in Hospital 🗆 Letter

🗆 Newspaper 🗆 Social media 🗆 Other ...............................................

Part 2 Medical Profile

14. Do you smoke? 🗆 No 🗆 Yes ..................... roll/day

1. Do you drink alcoholic beverages?

🗆 No

🗆 Yes (please inform kind of alcoholic beverage and amount of you drink per day)

............................................................................................................................

16. Have you ever had a physical examination, or a doctor diagnosed with various diseases?

*(Please check 🗸 in the box that applies to you. And answer all questions)*

| Disease | Never check | No | Yes |
| --- | --- | --- | --- |
| 1. Hypertension | 🗆 | 🗆 | 🗆 |
| 2. Diabetes | 🗆 | 🗆 | 🗆 |
| 3. Hyperlipidemia | 🗆 | 🗆 | 🗆 |
| 4. Heart disease | 🗆 | 🗆 | 🗆 |
| 5. Kidney disease | 🗆 | 🗆 | 🗆 |
| 6. Cancer .......................................................................... | 🗆 | 🗆 | 🗆 |
| 7. Asthma | 🗆 | 🗆 | 🗆 |
| 8. Obstructive Sleep Apnea: OSA | 🗆 | 🗆 | 🗆 |
| 9. Chronic Obstructive Pulmonary Disease: COPD | 🗆 | 🗆 | 🗆 |
| 10. Arthritis | 🗆 | 🗆 | 🗆 |
| 11. Other diseases .................................................... | 🗆 | 🗆 | 🗆 |

1. For those who are female Does your period come normally?

🗆 Menopause 🗆 Normally 🗆 Amenorrhea cause .......................................................

***************************************************************************************************************

Part 3 Behavior profile *(Please check 🗸 in the box that applies to you)*

1. How many main meals do you eat each day?

🗆 1 meal 🗆 2 meals 🗆 3 meals 🗆 more than 3 meals

1. Usually, the type of your breakfast is *(choose only answer is the behavior you tend to practice.)*

🗆 Not eat breakfast 🗆 Home cooking

🗆 Ready to eat food 🗆 Eating at the restaurant

1. Usually, the type of your food is (can choose more than one)

🗆 Home cooking 🗆 Microwave a frozen food

🗆 Eating at the restaurant 🗆 Buying food at local market

1. style of food that you usually eat (can choose more than one)

🗆 West fast food 🗆 Deep fried, Fritters or stir fried food

🗆 Food with coconut milk, butter 🗆 Boiled, steamed, baked, stewed food

🗆 Processed foods such as sausages and ham 🗆 Fermented food

🗆 Snacks 🗆 Bakery, bread, cakes, cookies

1. How many scoops of rice do you usually eat at each meal?
   *(1 ladle of rice equals about 5 tablespoons of rice.)*

🗆 less than or equal to 1 ladle 🗆 2 ladles 🗆 3 ladles 🗆 4 ladles 🗆 5 ladles 🗆 More than 5 ladles

1. Most sources of protein (e.g. meats, /egg/milk/tofu-soybean) which type do you eat?
   (can choose more than 1 answer)

🗆 Lean pork or lean chicken 🗆 Fish fillet 🗆 Eggs 🗆 Seafood

🗆 Belly pork or meat 🗆 animal offal

🗆 Processed meats such as sausages, ham, and sour pork

🗆 Milk (cow milk, goat milk) 🗆 Tofu, Soy products

1. How much meat do you eat per meal?

🗆 Less than or equal to 1 tablespoon per meal 🗆 2 tablespoons per meal

🗆 3 tablespoons per meal 🗆 more than 4 tablespoons per meal

1. How often do you eat vegetables?

🗆 Never eat 🗆 1 meal per day 🗆 2 meals per day 🗆 eat every meal

1. How many ladles of vegetables do you usually eat at each meal?

🗆 Less than or equal to 1 ladle 🗆 2 ladles

🗆 3 ladles 🗆 more than 3 ladles

1. How often do you eat fruits?

🗆 Never eat 🗆 1 meal per day 🗆 2 meals per day 🗆 eat every meal

1. How many portions of fruit do you usually eat at each meal? *(Set 1 portion of fruit equal to 1 medium guava or 1 medium banana or ½ medium banana or 2 medium oranges or rose apple, 4 rambutans or ½ mango or watermelon, papaya, pineapple about 8 pieces.)*

🗆 Never eat 🗆 Less than or equal to 1 portion per meal

🗆 2 portions per meal 🗆 3 portions per meal 🗆 more than 3 portions per meal

1. What kind of milk, yogurt, or dairy products do you prefer?

🗆 Never eat milk 🗆 plain milk 🗆 yogurt or yogurt drink

🗆 Flavored milk (eg. Chocolate, strawberry)

1. How often do you drink sugar-sweetened beverages (such as hot coffee with sugar, iced coffee, iced tea, or soft drinks)?

🗆 Never drink 🗆 1 time per day 🗆 2 – 3 times per day

🗆 more than 3 times per day 🗆 1 time per week 🗆 2 - 3 times per week

🗆 4 - 5 times per week

1. What do you think your current weight is?

🗆 Too underweight 🗆 Normal weight 🗆 Slightly heavier than before

🗆 Too overweight 🗆 too fat

1. What kind of behavior do you have? *(you can choose more than 1 answer)*

🗆 Likes sugary drinks, soft drinks, iced tea, ice coffee, fruit juices, or smoothies.

🗆 Need to eat a lot of rice per meal.

🗆 Eat a lot of fruit at a time (such as a kilo of rambutan, 3-4 oranges at a time).

🗆 Eat fast food often because they like it or have a rushed job.

🗆 Likes to eat sweets, snacks, bread, or bakery.

🗆 Add fish sauce, chili, fish sauce, and sugar to food regularly.

🗆 Likes to eat soup, curry soup, and noodle soup.

🗆 Likes to dip food with sauce ketchup chili sauce.

🗆 Likes to eat stir-fried food, deep-fried food, or curry with coconut milk.

🗆 Likes to eat nuts (eg. Peanut, cashew nut)

🗆 Likes to eat meat with fat, belly pork

1. Have you ever joined a weight loss program from a weight loss institute? or taken medication or dietary supplements during the past year the following? *(please put a mark 🗸 in front of the items that you have experienced in losing weight by that method.)*

🗆 No 🗆 Yes If yes, please fill out the details below. ⮷

• Have you ever received the services of a weight loss institute?

🗆 Marrie France 🗆 Body Shape 🗆 Yanhee hospital 🗆 Mhoo jai dee clinic

🗆 Pratunam Polyclinic 🗆 Skincare clinic 🗆 local clinic

🗆 other .....................................................

• Have you ever taken drugs or food products for weight loss?

🗆 Xenical 🗆 Reductil 🗆 Herbalife 🗆 Konyakky 🗆 Fitne

🗆 Hydrolite 🗆 The Cambridge Diet 🗆 Positrim 🗆 Herbal tea 🗆 Herbal supplement 🗆 Self-control diet 🗆 other .................................................................

• After receiving services or eating weight loss products, are you effective or not?

🗆 Ineffective 🗆 effective loss.......................kg in....................weeks ..................... months

Record form

Dietary in the past 24 hours (for 3 days)

ID....................................

“Record the food list. It is essential to know consumption behavior. So please record what you actually ate.”

**Recommendations for recording**

**1.** Record all foods including snacks and drinks you eat throughout the day. From the time you wake up until you go to bed (only the part you eat) by eating records on two non-adjacent weekdays (Mon, Tue, ...Friday or your working day) and one day off (Saturday or Sunday or your working day off).

**2**. Record food eaten both at home and away.

**3**. Meal Log by specifying the main meal (breakfast, lunch, dinner) or snacks and the time eaten.

**4**. Specify the place to eat, such as home, restaurant, workplace, friend's house, etc.

**5.** Record every food and drink eaten. and indicate how the food is cooked, e.g. roasted pork, fried chicken, stir-fried vegetables, Banana in syrup topped with coconut milk, etc.

**6.** Record the ingredients of the food. and amount of food eaten By specifying the size, volume and amount eaten, such as sweet and sour stir-fry Indicates eating 4 tablespoons of cucumber (or 1 ladle), 2 tablespoons of tomatoes, and 2 tablespoons of pork.

**7.** Record the drinks consumed. Is the volume or size and ingredients such as 1 medium bottle of cola or 280 cc., 1 cup of coffee, size 120 cc., put 2 teaspoons of coffee mate, 2 teaspoons of sugar, etc**.**

**8.** Example of a food journal

| Meals | Time | Place | menu/amount | cooking methods | ingredients | amount |
| --- | --- | --- | --- | --- | --- | --- |
| Morning | 6.30 am. | Home | 1 cup rice porridge | boiled | porridge | 2 ladles |
|  |  |  | ½ dish spicy dried shrimp salad | mixed | Dried shrimp | 2 tablespoons |
|  |  |  |  |  | sugar | 1 teaspoon |
|  |  |  | deep fried salted fish | fried | salted fish | 1 tablespoon |
|  |  |  | deep fried Chinese sausage | fried | Chinese sausage | 2 tablespoons |
|  |  |  |  |  | cooking oil | 1 tablespoon |
|  |  |  | 1 cup of coffee |  | coffee | 1 teaspoon |
|  |  |  |  |  | sugar | 2 teaspoons |
|  |  |  |  |  | whole milk  (Bear brand) | 2 tablespoon |
| mid lunch | 10.00 am. | work | 1 cup of coffee |  | coffee | 1 teaspoon |
|  |  |  |  |  | sugar | 2 teaspoons |
|  |  |  |  |  | coffee mate | 2 teaspoons |
| lunch | 12.00 pm | work | noodle with meatball and beef | boiled | noodle | 1 ladle |
|  |  |  | 1 bowl |  | bean sprout | 2 tablespoons |
|  |  |  | ate soup |  | beef | 1 teaspoon |
|  |  |  |  |  | meatball | 6 meatballs |
|  |  |  |  |  | fried garlic with oil | 2 teaspoons |
|  |  |  |  |  | sugar | 2 teaspoons |
|  |  |  | 3 rolls fresh spring rolls |  | spring roll pastry | 3 sheets |
|  |  |  |  |  | Chinese sausage | 2 teaspoons |
|  |  |  |  |  | tofu | 2 tablespoons |
|  |  |  |  |  | bean sprout | 2 tablespoons |
|  |  |  |  |  | crab meat | 1 tablespoon |
|  |  |  |  |  | sweet gravy | 1 tablespoon |
|  |  |  | 1 cup Banana in syrup topped with coconut milk | boiled | banana | 1 banana  cut to 4 pieces |
|  |  |  |  |  | coconut milk | 4 tablespoons |
|  |  |  |  |  | sugar | 2 teaspoon |
| mid dinner | 3 pm. | work | pineapple |  | 2 x 2 inch pineapple | 1 piece |
|  |  |  |  |  | a few of sugar mix with salt and chili powder |  |
| dinner | 6 pm. | home | 1 dish rice |  | rice | 2 ladle |
|  |  |  | 1 cup spicy chicken curry | boiled | chicken | 2 tablespoon |
|  |  |  |  |  | curry soup with coconut milk | 2 tablespoon |
|  |  |  |  |  | brinjal | 10 pieces |
|  |  |  | Stir Fried Kale with Salted Fish ½ dish | stir fried | kale | 4 tablespoon |
|  |  |  |  |  | salted fish | 2 tablespoon |
|  |  |  |  |  | cooking oil | 1 tablespoon |
|  |  |  | omelet with mince pork | fried | egg | ½ egg |
|  |  |  |  |  | cooking oil | 2 tablespoon |
|  |  |  |  |  | mince pork | 1 tablespoon |

Date record ................................................... ID ……………………………………. weight...............kg. height................cm.

food consume today is ( ) Normal day ( ) Weekend

| Meals | Time | Place | menu/amount | cooking methods | ingredients | amount |
| --- | --- | --- | --- | --- | --- | --- |
|  |  |  |  |  |  |  |
|  |  |  |  |  |  |  |
|  |  |  |  |  |  |  |
|  |  |  |  |  |  |  |
|  |  |  |  |  |  |  |
|  |  |  |  |  |  |  |
|  |  |  |  |  |  |  |
|  |  |  |  |  |  |  |
|  |  |  |  |  |  |  |
|  |  |  |  |  |  |  |
|  |  |  |  |  |  |  |
|  |  |  |  |  |  |  |
|  |  |  |  |  |  |  |
|  |  |  |  |  |  |  |
|  |  |  |  |  |  |  |
|  |  |  |  |  |  |  |
|  |  |  |  |  |  |  |
|  |  |  |  |  |  |  |
|  |  |  |  |  |  |  |
|  |  |  |  |  |  |  |
|  |  |  |  |  |  |  |
|  |  |  |  |  |  |  |
|  |  |  |  |  |  |  |
|  |  |  |  |  |  |  |
|  |  |  |  |  |  |  |
|  |  |  |  |  |  |  |
|  |  |  |  |  |  |  |
|  |  |  |  |  |  |  |
|  |  |  |  |  |  |  |

Questionnaires about daily life and exercise

Personal profile

Name..................................................................................... ID.................................

Gender male female Age....................year olds

Height...........................cm weight................................kg

congenital disease None Yes .................................................................

Daily life information

1. your career

government or state enterprise employee company employee

student other...........................................

2. What is the form of your work?

sit still standing throughout the work

sit and walk walking throughout work

3. How long on average do you spend sitting at work per day?

less than 10 minutes about 30 minutes 1 hour

more than 1 hour almost all day

4. How long on average do you spend standing at work per day?

less than 10 minutes about 30 minutes 1 hour

more than 1 hour almost all day

5. How long on average do you walk at work per day?

less than 10 minutes about 30 minutes 1 hour

more than 1 hour almost all day

Exercise information

1. When you have free time, what activities do you spend your free time on? *(more than 1 answer)*

exercise/play sports read a book watching television

listen to music travel phone call with friends

play social media other .....................................................

2. How often do you exercise or play sports?

every day every other day

3 days a week once a week

Weekend Didn't exercise or play sports at all.
