# Supplementary material for: Metabolic and inflammatory profiles, gut microbiota and lifestyle factors in overweight and normal weight young thai adults
Source: PLoS One. 2023 Jul 14;18(7):e0288286. doi: 10.1371/journal.pone.0288286 (PMC10348517; doi:10.1371/journal.pone.0288286)
Supplement: S3 File — (DOCX) [file pone.0288286.s004.docx]

แบบบันทึก การซักประวัติ และความถี่ในการรับประทานอาหาร

วัน/เดือน/ปี ที่บันทึก.....................................................

ส่วนที่ 1 ข้อมูลทั่วไป

1. ID...................................................................................................เพศ 🗆 ชาย 🗆 หญิง
2. น้ำหนักตัว ..............................กก. ส่วนสูง............................... ซม. ดัชนีมวลกาย .......................กก./ตรม. เส้นรอบเอว...................ซม (.......................นิ้ว) เส้นรอบสะโพก.......................ซม (........................นิ้ว)

ความดันโลหิต..........................มิลลิเมตรปรอท ชีพจร..........................ครั้งต่อนาที

1. ที่อยู่ปัจจุบัน บ้านเลขที่................................... ตรอก/ซอย........................................... ถนน...........................................ตำบล/แขวง..........................................อำเภอ/เขต...............................................จังหวัด.............................................รหัสไปรษณีย์.......................................
2. เบอร์โทรศัพท์ที่ติดต่อได้สะดวก เบอร์บ้าน................................................. เบอร์มือถือ........................................ เบอร์ที่ทำงาน...........................................
3. วัน/เดือน/ปีเกิด....................................................................อายุ.................................. ปี .............................. เดือน
4. เชื้อชาติ 🗆 ไทย 🗆 จีน 🗆 อื่นๆ.................................................................
5. ศาสนา 🗆 พุทธ 🗆 คริสต์ 🗆 อิสลาม 🗆 อื่นๆ...................................
6. สถานภาพสมรส 🗆 โสด 🗆 คู่ 🗆 หม้าย 🗆 หย่า 🗆 แยกกันอยู่

🗆 อื่นๆ.................................

1. มีบุตรหรือไม่ 🗆 มี จำนวน.....................คน 🗆 ไม่มี
2. ระดับการศึกษาสูงสุด

🗆 ไม่ได้เรียนหนังสือ 🗆 ประถมศึกษา 🗆 มัธยมศึกษา 🗆 ปวช, ปวส, อนุปริญญา

🗆 ปริญญาตรีหรือสูงกว่า 🗆 อื่นๆ.......................................................

1. อาชีพ

🗆 ไม่ได้ประกอบอาชีพ 🗆 รับราชการหรือรัฐวิสาหกิจ 🗆 พนักงานเอกชน หรือรับจ้าง

🗆 ประกอบธุรกิจส่วนตัว 🗆 อื่นๆ (โปรดระบุ).................................................................................

1. รายได้ของคุณโดยเฉลี่ยต่อเดือน

🗆 ต่ำกว่า 5,000 บาท 🗆 5,000 – 10,000 บาท 🗆 10,001 – 20,000 บาท

🗆 20,001 – 30,000 บาท 🗆 มากกว่า 30,000 บาท ขึ้นไป

1. ท่านรับทราบข้อมูลโครงการอบรมนี้ จากแหล่งข้อมูลใด

🗆 รายการวิทยุ 🗆 ป้ายโปสเตอร์ในร.พ.รามาธิบดี 🗆 จดหมายข่าวชมรม “กินให้สนุกฯ”

🗆 หนังสือพิมพ์ 🗆 อินเตอร์เน็ต / เว็บไซต์ 🗆 อื่นๆ โปรดระบุ...............................................

ส่วนที่ 2 ข้อมูลสุขภาพ

14 คุณสูบบุหรี่หรือไม่ 🗆 ไม่สูบ 🗆 สูบ.....................มวน ต่อวัน

1. คุณดื่มเครื่องดื่มที่มีแอลกอฮอล์หรือไม่

🗆 ไม่ดื่ม 🗆 ดื่ม (โปรดระบุชนิดและปริมาณที่ดื่มต่อวัน)................................................................

16 คุณเคยได้รับการตรวจร่างกายหรือวินิจฉัยจากแพทย์ว่าเป็นโรคต่างๆ ดังต่อไปนี้หรือไม่

*(โปรดกาเครื่องหมาย 🗸 ในช่องที่ตรงกับตัวคุณ และกรุณาตอบทุกข้อ)*

| โรค | ไม่เคยตรวจ | ไม่เป็น | เป็น |
| --- | --- | --- | --- |
| 1. ความดันโลหิตสูง | 🗆 | 🗆 | 🗆 |
| 2. เบาหวาน | 🗆 | 🗆 | 🗆 |
| 3. ไขมันในเลือดสูง | 🗆 | 🗆 | 🗆 |
| 4. โรคหัวใจ | 🗆 | 🗆 | 🗆 |
| 5. โรคไต | 🗆 | 🗆 | 🗆 |
| 6. โรคมะเร็ง ที่.......................................................................... | 🗆 | 🗆 | 🗆 |
| 7. โรคหอบหืด | 🗆 | 🗆 | 🗆 |
| 8. โรคหยุดหายใจขณะหลับ | 🗆 | 🗆 | 🗆 |
| 9. โรคหลอดลมปอดอุดกั้นเรื้อรัง | 🗆 | 🗆 | 🗆 |
| 10. โรคปวดข้อหรือไขข้ออักเสบ | 🗆 | 🗆 | 🗆 |
| 11. โรคอื่นๆที่แพทย์ตรวจพบ คือ..................................................... | 🗆 | 🗆 | 🗆 |

1. สำหรับท่านที่เป็นเพศหญิง ประจำเดือนของท่านมาตามปกติหรือไม่

🗆 หมดประจำเดือนแล้ว 🗆 มาปกติ 🗆 มาไม่ปกติ อย่างไร โปรดระบุ.......................................................

****************************************************************************************************************

ส่วนที่ 3 ข้อมูลพฤติกรรม *(โปรดกาเครื่องหมาย 🗸 ในช่องที่ตรงกับความคิดหรือพฤติกรรมของคุณ)*

1. คุณรับประทานอาหารมื้อหลัก วันละกี่มื้อ

🗆 1 มื้อ 🗆 2 มื้อ 🗆 3 มื้อ 🗆 มากกว่า 3 มื้อขึ้นไป

1. ส่วนใหญ่ อาหารมื้อเช้า ของคุณ .......... (ตอบข้อเดียวที่เป็นพฤติกรรมที่คุณมักจะปฏิบัติ)

🗆 ไม่รับประทานอาหารมื้อเช้า 🗆 ที่บ้านทำอาหารเองจากอาหารสด

🗆 ซื้ออาหารสำเร็จรูปมาอุ่นรับประทาน 🗆 ซื้อรับประทานนอกบ้าน

1. ส่วนใหญ่ อาหารที่คุณรับประทานเป็นแบบใด (ตอบได้มากกว่า 1 อย่าง)

🗆 ประกอบอาหารรับประทานเอง 🗆 อุ่นอาหารสำเร็จรูปรับประทาน

🗆 รับประทานอาหารตามร้านอาหาร 🗆 ซื้อรับประทานจากร้านค้าริมทาง

1. ประเภทอาหาร ที่คุณชอบรับประทานบ่อยๆ (ตอบได้มากกว่า 1 ข้อ)

🗆 อาหารฟาสต์ฟู้ดแบบตะวันตก 🗆 อาหารผัด ทอด ชุบแป้งทอด 🗆 อาหารที่ใส่กะทิ เนย

🗆 อาหารต้ม นึ่ง อบ ตุ๋น 🗆 อาหารแปรรูป เช่น ไส้กรอก แฮม 🗆 อาหารหมักดอง

🗆 ขนมขบเคี้ยว ขนมกรุบกรอบ 🗆 เบเกอรี่ ขนมปัง เค้ก คุกกี้

1. ส่วนใหญ่ คุณกินข้าวมื้อละ กี่ทัพพี (ข้าว 1 ทัพพี เท่ากับ ข้าวประมาณ 5 ช้อนโต๊ะ)

🗆 น้อยกว่าหรือเท่ากับ 1 ทัพพี 🗆 2 ทัพพี 🗆 3 ทัพพี 🗆 4 ทัพพี 🗆 5 ทัพพี 🗆 มากกว่า 5 ทัพพีขึ้นไป

1. ส่วนใหญ่ แหล่งของโปรตีน (เช่น เนื้อสัตว์ต่างๆ /ไข่/นม/เต้าหู้-ถั่วเหลือง) ที่คุณรับประทานคือประเภทใด (ตอบได้มากกว่า 1 ข้อ)

🗆 เนื้อหมูแดง เนื้อไก่ไม่ติดมัน 🗆 เนื้อปลา 🗆 ไข่ 🗆 อาหารทะเลต่างๆ

🗆 เนื้อติดมัน เนื้อสามชั้น 🗆 เครื่องในสัตว์ 🗆 เนื้อสัตว์แปรรูป เช่น ไส้กรอก แฮม แหนม

🗆 นมสด (นมวัว, นมแพะ) 🗆 เต้าหู้/ผลิตภัณฑ์จากถั่วเหลือง (เช่น นมถั่วเหลือง/ไวตามิลค์/แลตตาซอย)

1. ปริมาณเนื้อสัตว์ ที่คุณรับประทานต่อมื้อ มากแค่ไหน

🗆 น้อยกว่าหรือเท่ากับ 1 ช้อนกินข้าวต่อมื้อ 🗆 2 ช้อนกินข้าวต่อมื้อ

🗆 3 ช้อนกินข้าวต่อมื้อ 🗆 มากกว่า 4 ช้อนกินข้าวต่อมื้อ

1. ส่วนใหญ่ คุณรับประทานผัก บ่อยแค่ไหน

🗆 ไม่รับประทาน 🗆 1 มื้อต่อวัน 🗆 2 มื้อต่อวัน 🗆 รับประทานทุกมื้อ

1. ส่วนใหญ่ คุณรับประทานผัก มื้อละกี่ทัพพี

🗆 น้อยกว่าหรือเท่ากับ 1 ทัพพี 🗆 2 ทัพพี 🗆 3 ทัพพี 🗆 มากกว่า 3 ทัพพีขึ้นไป

1. ส่วนใหญ่ คุณรับประทานผลไม้ บ่อยแค่ไหน

🗆 ไม่รับประทาน 🗆 1 มื้อต่อวัน 🗆 2 มื้อต่อวัน 🗆 รับประทานทุกมื้อ

1. ส่วนใหญ่ คุณรับประทานผลไม้มื้อละกี่ส่วน (กำหนดให้ 1 ส่วน ของผลไม้ เท่ากับ ฝรั่ง 1 ผลกลาง หรือ กล้วยน้ำว้า 1 ผลกลาง หรือ กล้วยหอม ½ ผลกลาง หรือ ส้ม 2 ผลกลาง หรือ ชมพู่ เงาะ 4 ผล หรือ มะม่วง ½ ผล หรือ แตงโม มะละกอ สับปะรด ประมาณ 8 ชิ้นพอคำ)

🗆 ไม่รับประทาน 🗆 น้อยกว่าหรือเท่ากับ 1 ส่วนต่อมื้อ 🗆 2 ส่วนต่อมื้อ

🗆 3 ส่วนต่อมื้อ 🗆 มากกว่า 3 ส่วนต่อมื้อ

1. คุณเลือกดื่มนม นมถั่วเหลือง โยเกิร์ต หรือผลิตภัณฑ์ที่ทำจากนม แบบใด

🗆 ไม่ดื่มนม 🗆 นมรสจืด 🗆 นมเปรี้ยว ยาคูลท์ 🗆 นมรสหวาน เช่น รสช็อคโกแลต รสสตรอเบอรี่ ฯลฯ

1. คุณดื่มเครื่องดื่มรสหวาน เช่น กาแฟร้อนใส่น้ำตาล กาแฟเย็น ชาเย็น น้ำอัดลม น้ำหวานที่ตักขาย บ่อยแค่ไหน

🗆 ไม่ดื่ม 🗆 วันละ 1 ครั้ง 🗆 วันละ 2 – 3 ครั้ง 🗆 วันละมากกว่า 3 ครั้ง

🗆 1 ครั้งต่อสัปดาห์ 🗆 2 - 3 ครั้งต่อสัปดาห์ 🗆 4 - 5 ครั้งต่อสัปดาห์

1. คุณคิดว่า น้ำหนักตัวในปัจจุบัน เป็นอย่างไร

🗆 น้ำหนักน้อยมาก 🗆 น้ำหนักพอดี 🗆 น้ำหนักมากกว่าแต่ก่อนเล็กน้อย

🗆 น้ำหนักมากเกินไปแล้ว 🗆 อ้วนมาก

1. คุณมีพฤติกรรมแบบไหน (ตอบได้มากกว่า 1 ข้อ)

🗆 ชอบดื่มน้ำหวาน น้ำอัดลม ชาเย็น กาแฟเย็น น้ำผลไม้คั้นหรือปั่น 🗆 ต้องกินข้าวเยอะๆ จะได้ไม่หิว

🗆 กินผลไม้ครั้งละมากๆ (เช่น เงาะเป็นกิโล ส้มครั้งละ 3-4 ลูก) 🗆 กินฟาสต์ฟู้ดบ่อย เพราะชอบหรือมีงานรีบเร่ง

🗆 ชอบกินขนมหวาน ขนมขบเคี้ยว ขนมปัง เบเกอรี่ต่างๆ 🗆

🗆 เติมน้ำปลา พริกน้ำปลา น้ำตาล ในอาหารเป็นประจำ 🗆 ชอบซดน้ำแกง น้ำซุบ น้ำก๋วยเตี๋ยว จนหมด

🗆 ชอบเติมน้ำจิ้ม ซอสมะเขือเทศ ซอสพริก ฯลฯ 🗆 กินอาหารผัดๆ ทอดๆ หรือแกงกะทิ บ่อย

🗆 ชอบกินถั่วต่างๆ (เช่น ถั่วลิสง เม็ดมะม่วงหิมพานต์) 🗆 ชอบกินเนื้อสัตว์ติดมัน ติดหนัง

1. ท่านเคยเข้าร่วมโปรแกรมการลดน้ำหนักจากสถาบันลดน้ำหนัก หรือรับประทานยา หรือผลิตภัณฑ์อาหารเสริมในช่วงปีที่ผ่านมา ดังต่อไปนี้หรือไม่ (โปรดกา เครื่องหมาย 🗸 หน้าข้อ ที่ท่านเคยมีประสบการณ์ลดน้ำหนักโดยวิธีนั้นๆ)

🗆 ไม่เคย

🗆 เคย ถ้าตอบว่าเคย กรุณากรอกรายละเอียดด้านล่างนี้ ⮷

- ท่านเคยเข้ารับบริการของสถาบันลดน้ำหนัก

🗆 Marrie France 🗆 Body Shape 🗆 โรงพยาบาลยันฮี 🗆 คลินิกหมอใจดี

🗆 ประตูน้ำโพลีคลินิก 🗆 คลินิกโรคผิวหนัง 🗆 คลินิกทั่วไปใกล้บ้าน 🗆 อื่นๆ ระบุ............................

- ท่านเคยรับประทานยาหรือผลิตภัณฑ์อาหารสำหรับลดน้ำหนัก

🗆 Xenical 🗆 Reductil 🗆 เฮอร์บาไลฟ์ (Herbalife) 🗆 คอนยัคกี้

🗆 ฟิตเน่ 🗆 ไฮโดรไลท์ 🗆 เคมบริดไดเอ็ท 🗆 โพซิทริม

🗆 ชาสมุนไพร ชาลดน้ำหนัก 🗆 ยาสมุนไพร 🗆 ควบคุมอาหารด้วยตนเอง

🗆 อื่นๆ โปรดระบุ.................................................................

- หลังจากเข้ารับบริการหรือรับประทานผลิตภัณฑ์ลดน้ำหนัก ท่านได้ผล หรือไม่

🗆 ไม่ได้ผล 🗆 ได้ผล ลดได้.......................กิโลกรัม ภายใน....................สัปดาห์.....................เดือน

แบบบันทึกข้อมูล

รายการอาหารที่รับประทานในรอบ 24 ชั่วโมง (สำหรับ 3 วัน)

รหัส....................................

“บันทึกรายการอาหาร เป็นสิ่งจำเป็นสำหรับการทราบพฤติกรรมการบริโภค ดังนั้นขอให้บันทึกสิ่งที่รับประทานจริง”

# ข้อแนะนำในการบันทึก

**1.** บันทึกอาหารทุกชนิดรวมทั้งขนมและเครื่องดื่มที่ท่านรับประทานตลอดวัน ตั้งแต่ท่านตื่นนอน จนเข้านอน (เฉพาะส่วนที่ท่านรับประทาน) **โดยบันทึกการรับประทาน ในวันธรรมดาสองวัน ที่ไม่ติดกัน (จันทร์, อังคาร, ....ศุกร์ หรือวันทำงานของคุณ) และวันหยุดหนึ่งวัน (เสาร์ หรืออาทิตย์ หรือวันหยุดทำงานของคุณ)**

**2.** บันทึกอาหารที่รับประทานทั้งที่บ้านและนอกบ้าน

**3.** บันทึกมื้ออาหาร โดยระบุมื้อหลัก (เช้า กลางวัน เย็น) หรือ อาหารว่าง และเวลาที่รับประทาน

**4.** ระบุสถานที่กิน เช่น บ้าน ร้านอาหาร ที่ทำงาน บ้านเพื่อน ฯลฯ

**5.** บันทึกอาหารและเครื่องดื่มทุกชนิดที่กิน และระบุว่าอาหารนั้นปรุงอย่างไร เช่น หมูย่าง ไก่ทอด ผัดผัก

กล้วยเชื่อมราดกะทิ ฯลฯ

**6.** บันทึกเครื่องประกอบของอาหาร และปริมาณอาหารที่กิน โดยระบุขนาดปริมาตรและจำนวนที่กิน เช่น ผัดเปรี้ยวหวาน ให้ระบุว่ากินแตงกวา 4 ช้อนโต๊ะ (หรือ 1 ทัพพี) มะเขือเทศ 2 ช้อนโต๊ะ เนื้อหมู 2 ช้อนโต๊ะ

**7.** บันทึกเครื่องดื่มที่กิน เป็นปริมาตรหรือขนาด และส่วนผสม เช่น โคล่า 1 ขวดกลาง หรือ 280 ซี.ซี. กาแฟ 1 ถ้วย ขนาด 120 ซี.ซี. ใส่คอฟฟี่เมต 2 ช้อนชา น้ำตาลทราย 2 ช้อนชา เป็นต้น

**8.** ตัวอย่างการบันทึกอาหาร

| มื้ออาหาร | เวลา | สถานที่ | ชื่ออาหาร/ปริมาณ | วิธีปรุง | ส่วนประกอบของอาหาร | ปริมาณ |
| --- | --- | --- | --- | --- | --- | --- |
| เช้า | 6.30 น. | บ้าน | ข้าวต้ม 1 ถ้วย | ต้ม | ข้าวต้ม (คิดเฉพาะเนื้อข้าว) | 2 ทัพพี |
|  |  |  | ยำกุ้งแห้ง ½ จาน | ยำ | กุ้งแห้ง | 2 ช้อนโต๊ะ |
|  |  |  |  |  | น้ำตาล | 1 ช้อนชา |
|  |  |  | ปลาเค็มทอด | ทอด | ปลาเค็ม | 1 ช้อนโต๊ะ |
|  |  |  | กุนเชียงทอด | ทอด | กุนเชียง | 2 ช้อนโต๊ะ |
|  |  |  |  |  | น้ำมันกุ๊ก (ที่ใช้ทอด) | 1 ช้อนโต๊ะ |
|  |  |  | กาแฟ 1 แก้ว |  | กาแฟ | 1 ช้อนชา |
|  |  |  |  |  | น้ำตาล | 2 ช้อนชา |
|  |  |  |  |  | นมสด (ตราหมี) | 2 ช้อนโต๊ะ |
| อาหารว่างเช้า | 10.00 น. | ที่ทำงาน | กาแฟ 1 แก้ว |  | กาแฟ | 1 ช้อนชา |
|  |  |  |  |  | น้ำตาล | 2 ช้อนชา |
|  |  |  |  |  | คอฟฟี่เมต | 2 ช้อนชา |
| กลางวัน | 12.00 น. | ที่ทำงาน | ก๋วยเตี๋ยวลูกชิ้นเนื้อสด | ต้ม | เส้นก๋วยเตี๋ยว | 1 ทัพพี |
|  |  |  | 1 ชาม |  | ถั่วงอก | 2 ช้อนโต๊ะ |
|  |  |  | กินน้ำก๋วยเตี๋ยวหมด |  | เนื้อสด | 1 ช้อนชา |
|  |  |  |  |  | ลูกชิ้น | 6 ลูก |
|  |  |  |  |  | กระเทียมเจียว | 2 ช้อนชา |
|  |  |  |  |  | น้ำตาลทราย | 2 ช้อนชา |
|  |  |  | ปอเปี๊ยะสด 3 ชิ้น |  | แป้งปอเปี๊ยะ | 3 แผ่น |
|  |  |  |  |  | กุนเชียง | 2 ช้อนชา |
|  |  |  |  |  | เต้าหู้ | 2 ช้อนโต๊ะ |
|  |  |  |  |  | ถั่วงอก | 2 ช้อนโต๊ะ |
|  |  |  |  |  | เนื้อปู | 1 ช้อนโต๊ะ |
|  |  |  |  |  | น้ำราด (รสออกค่อนข้างหวาน) | 1 ช้อนโต๊ะ |
|  |  |  | กล้วยบวดชี 1 ถ้วย | ต้ม | กล้วยน้ำว้า (1 ลูกผ่า 4 ชิ้น) | 4 ชิ้น |
|  |  |  |  |  | กะทิ | 4 ช้อนโต๊ะ |
|  |  |  |  |  | น้ำตาล | 2 ช้อนชา |
| อาหารว่างบ่าย | 15.00 น. | ที่ทำงาน | สับปะรด |  | สับปะรดขนาด 2 x 2 นิ้ว | 1 ชิ้น |
|  |  |  |  |  | พริกกับเกลือ, น้ำตาล เล็กน้อย |  |
| มื้ออาหาร | เวลา | สถานที่ | ชื่ออาหาร/ปริมาณ | วิธีปรุง | ส่วนประกอบของอาหาร | ปริมาณ |
| เย็น | 18.00 น. | ที่บ้าน | ข้าวสวย 1 จาน |  | ข้าวสวย | 2 ทัพพี |
|  |  |  | แกงเผ็ดไก่ 1 ถ้วย | ต้ม | เนื้อไก่ | 2 ช้อนโต๊ะ |
|  |  |  | (กินน้ำแกงกะทิด้วย) |  | น้ำแกง (กะทิ) | 2 ช้อนโต๊ะ |
|  |  |  |  |  | มะเขือเปราะ (ผ่า 4 ) | 10 ชิ้น |
|  |  |  | ผัดคะน้าปลาเค็ม ½ จาน | ผัด | คะน้า | 4 ช้อนโต๊ะ |
|  |  |  |  |  | ปลาเค็ม | 2 ช้อนโต๊ะ |
|  |  |  |  |  | น้ำมันกุ๊ก | 1 ช้อนโต๊ะ |
|  |  |  | ไข่เจียวหมูสับ | ทอด | ไข่ | ½ ฟอง |
|  |  |  |  |  | น้ำมันกุ๊ก | 2 ช้อนโต๊ะ |
|  |  |  |  |  | หมูสับ | 1 ช้อนโต๊ะ |

วันที่จดบันทึก ................................................... รหัส…………………………………….

น้ำหนัก...............กก. ส่วนสูง................ซ.ม. อาหารที่รับประทานวันนี้ ( ) วันปกติ ( ) วันหยุด

| มื้ออาหาร | เวลา | สถานที่ | ชื่ออาหาร/ปริมาณ | วิธีปรุง | ส่วนประกอบของอาหาร | ปริมาณ |
| --- | --- | --- | --- | --- | --- | --- |
|  |  |  |  |  |  |  |
|  |  |  |  |  |  |  |
|  |  |  |  |  |  |  |
|  |  |  |  |  |  |  |
|  |  |  |  |  |  |  |
|  |  |  |  |  |  |  |
|  |  |  |  |  |  |  |
|  |  |  |  |  |  |  |
|  |  |  |  |  |  |  |
|  |  |  |  |  |  |  |
|  |  |  |  |  |  |  |
|  |  |  |  |  |  |  |
|  |  |  |  |  |  |  |
|  |  |  |  |  |  |  |
|  |  |  |  |  |  |  |
|  |  |  |  |  |  |  |
|  |  |  |  |  |  |  |
|  |  |  |  |  |  |  |
|  |  |  |  |  |  |  |
|  |  |  |  |  |  |  |
|  |  |  |  |  |  |  |
|  |  |  |  |  |  |  |
|  |  |  |  |  |  |  |
|  |  |  |  |  |  |  |
|  |  |  |  |  |  |  |
|  |  |  |  |  |  |  |
|  |  |  |  |  |  |  |
|  |  |  |  |  |  |  |
|  |  |  |  |  |  |  |

แบบสอบถามเกี่ยวกับการใช้ชีวิตประจำวันและการออกกำลังกาย

ข้อมูลส่วนตัว

ชื่อ-นามสกุล......................................................................................................................

เพศ ชาย หญิง อายุ....................ปี

ส่วนสูง...........................เซนติเมตร น้ำหนัก................................กิโลกรัม

โรคประจำตัว ไม่มี มี ระบุ.................................................................

ข้อมูลการใช้ชีวิตประจำวัน

1. อาชีพของท่าน

ข้าราชการ/พนักงานรัฐวิสาหกิจ พนักงานเอกชน/รับจ้าง

นิสิต/นักศึกษา อื่นๆ ระบุ............................................

2. การทำงานของท่านเป็นรูปแบบใด

นั่งอยู่กับที่ ยืนตลอดการทำงาน

นั่งสลับเดิน เดินตลอดการทำงาน

3. ท่านใช้เวลานั่งในที่ทำงานโดยเฉลี่ยนานเท่าใดต่อวัน

น้อยกว่า 10 นาที ประมาณ 30 นาที 1 ชั่วโมง

มากกว่า 1 ชั่วโมง เกือบทั้งวัน

4. ท่านใช้เวลายืนในที่ทำงานโดยเฉลี่ยนานเท่าใดต่อวัน

น้อยกว่า 10 นาที ประมาณ 30 นาที 1 ชั่วโมง

มากกว่า 1 ชั่วโมง เกือบทั้งวัน

5. ท่านใช้เวลาเดินในที่ทำงานโดยเฉลี่ยนานเท่าใดต่อวัน

น้อยกว่า 10 นาที ประมาณ 30 นาที 1 ชั่วโมง

มากกว่า 1 ชั่วโมง เกือบทั้งวัน

ข้อมูลการออกกำลังกาย

1. เมื่อมีเวลาว่างท่านใช้เวลาว่างประกอบกิจกรรมใด (ตอบได้มากกว่า 1 ข้อ)

ออกกำลังกาย/เล่นกีฬา อ่านหนังสือ ดูโทรทัศน์

ฟังเพลง ท่องเที่ยว โทรศัพท์คุยกับเพื่อน

เล่นsocial media อื่นๆ ระบุ.....................................................

2. ท่านออกกำลังกายหรือเล่นกีฬาบ่อยครั้งแค่ไหน

ทุกวัน วันเว้นวัน

สัปดาห์ละ 3 วัน สัปดาห์ละครั้ง

เฉพาะเสาร์-อาทิตย์ ไม่ได้ออกกำลังกายหรือเล่นกีฬาเลย
